# Gender-Specific Genetic Predisposition to Breast Cancer: *BRCA* Genes and Beyond

**DOI:** 10.3390/cancers16030579

**Published:** 2024-01-30

**Authors:** Virginia Valentini, Agostino Bucalo, Giulia Conti, Ludovica Celli, Virginia Porzio, Carlo Capalbo, Valentina Silvestri, Laura Ottini

**Affiliations:** 1 Department of Molecular Medicine, Sapienza University of Rome, 00161 Rome, Italy; virginia.valentini@uniroma1.it (V.V.); agostino.bucalo@uniroma1.it (A.B.); giulia.conti@uniroma1.it (G.C.); ludovica.celli@uniroma1.it (L.C.); virginia.porzio@uniroma1.it (V.P.); carlo.capalbo@uniroma1.it (C.C.); valentina.silvestri@uniroma1.it (V.S.); 2 Medical Oncology Unit, Sant’Andrea University Hospital, 00189 Rome, Italy

**Keywords:** breast cancer, male breast cancer, gender oncology, *BRCA*, *PALB2*, *CHEK2*, *ATM*, PRS, risk assessment, personalized clinical management

## Abstract

**Simple Summary:**

The role of gender in oncology is an issue that is starting to be recognized as being of extreme importance in the last few years. While breast cancer is commonly perceived as a female-only disease, it does also occur in men, although rarely, thus opening relevant gender issues. Breast cancer in men is much less studied, with most knowledge coming from research on female breast cancer; however, several crucial differences have begun to be discovered between male and female patients. For example, the gender-specific impact and magnitude of risks conferred by breast cancer genetic risk factors are emerging, and they should be taken into consideration for a proper personalized clinical management. Overall, addressing all the challenges and the open issues regarding breast cancer genetic predisposition, including gender, will have an important clinical impact on the management of patients of both sexes.

**Abstract:**

Among neoplastic diseases, breast cancer (BC) is one of the most influenced by gender. Despite common misconceptions associating BC as a women-only disease, BC can also occur in men. Additionally, transgender individuals may also experience BC. Genetic risk factors play a relevant role in BC predisposition, with important implications in precision prevention and treatment. The genetic architecture of BC susceptibility is similar in women and men, with high-, moderate-, and low-penetrance risk variants; however, some sex-specific features have emerged. Inherited high-penetrance pathogenic variants (PVs) in *BRCA1* and *BRCA2* genes are the strongest BC genetic risk factor. *BRCA1* and *BRCA2* PVs are more commonly associated with increased risk of female and male BC, respectively. Notably, *BRCA*-associated BCs are characterized by sex-specific pathologic features. Recently, next-generation sequencing technologies have helped to provide more insights on the role of moderate-penetrance BC risk variants, particularly in *PALB2*, *CHEK2,* and *ATM* genes, while international collaborative genome-wide association studies have contributed evidence on common low-penetrance BC risk variants, on their combined effect in polygenic models, and on their role as risk modulators in *BRCA1/2* PV carriers. Overall, all these studies suggested that the genetic basis of male BC, although similar, may differ from female BC. Evaluating the genetic component of male BC as a distinct entity from female BC is the first step to improve both personalized risk assessment and therapeutic choices of patients of both sexes in order to reach gender equality in BC care. In this review, we summarize the latest research in the field of BC genetic predisposition with a particular focus on similarities and differences in male and female BC, and we also discuss the implications, challenges, and open issues that surround the establishment of a gender-oriented clinical management for BC.

## 1. Introduction

Gender-sensitive medicine is an innovative approach that takes into consideration the impact of differences in biological sex or gender identity on health and disease status, with the goal of improving prevention, screening, diagnosis, and treatment of all individuals [1].

The importance of gender in oncology is starting to be recognized, but this issue is still undervalued if compared to other medical disciplines [1]. The interplay between genetic mediators, hormonal mediators, including estrogens, progesterone and androgens, age, and reproductive status may modulate both local determinants of carcinogenesis, such as cancer-initiating cells and the tumor microenvironment, and systemic ones, such as cell metabolism and the immune system [2].

It is noteworthy that all these aforementioned features are largely involved in the pathogenesis of breast cancer (BC). Indeed, among neoplastic diseases, BC is one of the most influenced by gender. Although often mistakenly considered as a female-only disease, BC may also occur in men (referring to cisgender men) [3,4].

Moreover, BC may also occur in transgender individuals, both female-to-male (transgender men) and male-to-female (transgender women), although with a lower incidence compared with women (referring to cisgender women) [5].

Cancer arising in female breast tissue (female breast cancer, FBC) is the most common cancer and one of the leading causes of death among women globally [6], while cancer arising in male breast tissue (male breast cancer, MBC) is a rare disease, representing less than 1% of all cancers in men and of all BCs [3]. However, MBC incidence has been increasing over the last 30 years, and morbidity in MBC patients is a serious concern [7,8].

The rarity of MBC has precluded the development of ad hoc clinical trials, and currently, clinical management and therapeutic options of MBC patients is informed almost entirely by FBC research [9]. Notably, although an improvement in BC survival was observed in the last decades, mortality after cancer diagnosis is higher among male patients with BC compared with their female counterparts, even after accounting for possible prognostic factors, suggesting that such disparity may be due to factors yet to be identified [10,11]. Indeed, although MBC is thought to resemble post-menopausal FBC, increasing evidence indicates that MBC may be different, with unique molecular features, suggesting sex-specific differences in terms of biological and clinical behavior [12,13,14].

BC in both sexes is likely to be caused by the concurrent effects of different risk factors, including advanced age, BC family history, increased levels of estrogens (i.e., estradiol), and environmental exposures to carcinogens [15,16,17]. Clearly, the molecular and epidemiological determinants of MBC are not confounded by reproductive-history-related variables as in FBC [3,8].

Overall, it is estimated that 10–20% of BC cases are associated with hereditary factors [18]. Exploration into the complex BC genetic predisposition might be facilitated more by MBC, unencumbered by the many confounding factors that make FBC a heterogeneous disease. Nevertheless, sex-specific differences in the impact and magnitude of risks conferred by BC genetic risk factors are emerging, and they should be taken into consideration for a proper personalized clinical management.

This review covers all the main genes and genetic variants associated with BC risk, highlighting differences and similarities between BC genetic predisposition in the two sexes, as well as addressing the implications, challenges, and open issues of establishing a gender-oriented BC clinical management.

## 2. Methods

To summarize and describe the latest research in the field of BC genetic predisposition with particular focus on similarities and differences in male and female BC, we conducted a systematic literature search by using PubMed (http://www.ncbi.nlm.nih.gov/pubmed/ accessed up to 20 December 2023). We selected original articles and reviews in English, published up to December 2023. The following search key words were used to query the PubMed website: ‘breast cancer and gender’, ‘breast cancer and genetic predisposition’, ‘breast cancer and BRCA’, ‘male breast cancer and genetic predisposition’, ‘male breast cancer and BRCA’, ‘breast cancer genetic predisposition and transgender’, and ‘breast cancer management’. The abstracts resulting from these queries were individually assessed, and inclusion in the review was evaluated based on relevance. Exclusion criteria were papers written in non-English languages, case-reports, low-quality studies, and articles evaluated as out of the review’s scope.

## 3. The Genetic Architecture of BC Predisposition

The genetic architecture of BC predisposition may be explained by genetic risk factors that, based on their frequency and the magnitude of their impact on BC susceptibility, can be classified as high-, moderate-, and low-penetrance.

About 30 years ago, linkage analyses performed in families affected by multiple cases of FBC allowed for the identification of *BRCA1*, the first high-penetrance gene associated with BC susceptibility [19]. Shortly after, *BRCA2* was identified, analyzing families affected by both female and male BC [20].

Most cases of hereditary breast and ovarian cancer (HBOC) syndrome are linked to *BRCA1* or *BRCA2* genes. Germline pathogenic and likely pathogenic variants (herein called pathogenic variants, PVs) in these genes can be found in about 25% of families with HBOC [21].

The role of *BRCA1* and *BRCA2* PVs in BC susceptibility is significantly different in the two sexes, with *BRCA1* mainly involved in females while *BRCA2* in males. PVs in *BRCA2* are often found in patients with MBC who have multiple cases of BC/ovarian cancer (OC) in their family, but they have also been found in patients with MBC without family history [3]. Notably, a high percentage of BC patients without *BRCA1*/2 PVs was shown to have a positive family history of BC, suggesting the existence of other susceptibility factors [22].

Direct sequencing of candidate genes involved in *BRCA1*/2-associated DNA damage repair pathways led to the identification of other BC susceptibility genes, including *PALB2*, *CHEK2,* and *ATM*. In the last years, next-generation sequencing (NGS) analyses performed in BC by multigene panel testing have given the opportunity to identify PVs in a large number of candidate BC susceptibility genes, as well as to clarify their role and impact on BC predisposition [23,24]. These genes were generally classified as moderate-penetrance genes since their PVs confer a smaller risk of BC than *BRCA1*/2 PVs. On the other hand, a small but increasing number of studies applied multigene panel testing to investigate additional genes associated with MBC predisposition [25,26,27,28,29,30,31,32]. Collaborative studies are starting to provide reliable gender-specific cancer risk estimates for BC susceptibility genes [32,33,34].

BCs unaccounted by PVs in currently known BC susceptibility genes can be explained by the occurrence of low-penetrance risk variants [35]. A polygenic model, in which many variants that confer low risk individually act in combination to confer much larger risk in the population, has been suggested for susceptibility to several types of cancer, including BC. This hypothesis has been confirmed for both female and male BC by international multigroup collaborations working in genome-wide association studies (GWAS) [36,37,38,39,40,41,42,43,44]. Overall, the genetic architecture of BC predisposition is similar in both sexes; however, some differences in the impact of the risk conferred by specific genes have emerged (Table 1).

### 3.1. BRCA1

*BRCA1*, the first gene identified in 1994 as responsible for HBOC syndrome [19], is located on the long arm of chromosome 17 and encodes for an 1863 amino acid protein. This protein, expressed in a wide range of tissues, is critical in the DNA damage repair mechanism, cell cycle regulation, and other functions, such as transcriptional regulation and protein degradation by ubiquitination [55].

Germline PVs of *BRCA1* are reported in 1–7% of FBC patients unselected for family history or age at onset [56]. The largest available prospective study showed that women with germline *BRCA1* PVs have a cumulative BC risk to age 80 years of 72%, compared with the 13% of the general female population [45]. Risk assessment based on familial cases provided risk estimates higher than 80% [46], whereas recent population-based studies provided a lifetime risk estimate around 50% [23,24] (Table 1).

Prospective studies are not available for male *BRCA1* PV carriers; however, recent risk assessment, based on pedigree data, showed that the cumulative BC risk at age 80 years is around 0.4% for male *BRCA1* PV carriers, fourfold higher than the risk of 0.1% reported for the general male population [34]. Overall, compared with FBC, *BRCA1* PVs are quite rare in MBC cases, accounting for up to 4% of unselected MBC cases, but are more frequent in specific populations in which a founder effect is known to occur, representing 10–16% of MBC cases [46,57].

A large number of loss-of-function PVs, such as nonsense, small insertions or deletions, or splice or large rearrangements, occurring in functional domains are described along the whole *BRCA1* gene and three BC cluster regions (BCCRs), located at c.179 to c.505, c.4328 to c.4945, and c. 5261 to c.5563, were identified [58]. Large-scale rearrangements, including insertions, deletions, or duplications of more than 500 kb of DNA, have been also identified in BC cases [59,60,61].

Specific *BRCA1* PVs show high frequency in specific countries or ethnic groups, particularly in genetically isolated populations, and are in part responsible for the variability in BC incidence rates among countries [62]. For example, the founder *BRCA1* PVs c.68_69delAG and c.5266dupC in the Ashkenazi Jewish female and male BC patients account for a significant portion of all familial BC in this population [63,64], and several other founder *BRCA1* PVs have been observed in different populations worldwide [65,66].

It is now well established that *BRCA1*-related FBCs represent a subgroup of tumors characterized by a peculiar phenotype characterized by lack of estrogen receptor (ER), progesterone receptor (PR), and human epidermal growth factor receptor 2 (HER2) and defined as triple negative BC (TNBC) [67]; in addition, these tumors are more frequently of high grade compared with sporadic tumors [68].

By contrast, the phenotypic correlation between *BRCA1* PVs and TNBC observed in women was not observed in men. It was shown that MBCs with germline PVs in the *BRCA1* gene were more likely to be ER positive; PR positive; and, overall, non-TNBC compared with *BRCA1* FBCs [12]. Drivers of hypoxia and related proteins associated with *BRCA1* FBCs were shown to have a marginal role in MBCs [69].

In addition to BC, *BRCA1* is a well-established OC susceptibility gene, with a cumulative risk to age 80 years of 44% for female *BRCA1* PV carriers [45]; moreover, *BRCA1* PVs were recently associated with a twofold increased risk of pancreatic and stomach cancers in both sexes [34].

### 3.2. BRCA2

The *BRCA2* gene, identified soon after *BRCA1* in 1995, is located on the long arm of chromosome 13 and encodes for a 3418 amino acid protein [20]. This protein is expressed in response to cell proliferation, and its expression is initiated before DNA synthesis [70].

Both *BRCA1* and *BRCA2* are involved in maintaining genome integrity by engaging in DNA repair, cell cycle checkpoint control, and the regulation of key mitotic or cell division steps [55]. While *BRCA1* is a pleiotropic DNA damage response protein that functions in both checkpoint activation and DNA repair, *BRCA2* is a mediator of the core mechanism of homologous recombination (HR) [70].

Germline PVs of *BRCA2* are reported in 1–3% of FBC patients unselected for family history or age at onset [56]. The largest available prospective study showed that women with germline *BRCA1* PVs have a cumulative BC risk to age 80 years of 69% [45], whereas recent population-based studies provided a lifetime risk estimate around 50%, similar to the risk reported for *BRCA1* PVs [23,24] (Table 1).

Initial studies reported that the majority (81%) of the HBOC families were due to *BRCA1* PVs; conversely, most families with both male and female BCs were due to *BRCA2* (76%) [46]. Indeed, inherited PVs in *BRCA2* are the strongest genetic risk factor for MBC [71]. The estimated lifetime risk of MBC in *BRCA2* PV carriers is about 4%, more than 40-fold higher than the risk of 0.1% reported in the general male population [34]. Notably, male *BRCA2* PVs carriers are significantly more likely to develop cancer, particularly BC, and are at increased risk of developing second breast and non-breast tumors, compared to male *BRCA1* PVs carriers [72].

As in *BRCA1*, loss-of-function *BRCA2* PVs are identified along the whole gene, although several putative BCCRs have been defined, particularly at the 3′ end of the gene [58]. Like *BRCA1*, specific founder *BRCA2* PVs are also present in genetically isolated population groups, as for example, the Ashkenazi Jewish founder *BRCA2* c.5964delT [62]. In Icelanders, the predominant *BRCA2* c.771_775del accounts for a high proportion of BC families and was detected in up to 40% of MBC cases [73]. Overall, in high-risk families, or in populations in which a founder effect was observed, PVs in the *BRCA2* gene are estimated to be responsible for 60–76% of MBCs [74]. Interestingly, large genomic rearrangements in *BRCA2* are more frequent in families with MBC [61,75,76,77], and, on the other hand, *BRCA2* rearrangements seem to be infrequent in MBC cases unselected for family history [78].

It has been shown that FBC associated with *BRCA2* PVs are more similar to sporadic tumors, exhibiting a luminal phenotype characterized by ER and PR overexpression, and they are often HER2 negative [67,79]. By contrast, MBCs associated with *BRCA2* PVs display specific pathologic features suggestive of an aggressive phenotype, such as higher histologic grade, compared both with FBC in *BRCA2* PV carriers and with MBC in the general population [12,80,81]. In particular, high histologic grade breast tumors are more frequent among male *BRCA2* PV carriers diagnosed at younger ages (<50 years) than among those diagnosed at older ages [12]. Moreover, *BRCA2*-associated MBCs displayed a higher TNM status, an over-representation of invasive micropapillary, and a lower representation of lobular morphologies, compared with *BRCA2*-associated FBCs [81,82].

Overall, *BRCA2* has been associated with a more heterogeneous cancer spectrum, compared with *BRCA1*. Similar to *BRCA1*, *BRCA2* is also an OC susceptibility gene, with a cumulative risk to age 80 years of 17% for female *BRCA2* PV carriers [45]. Moreover, *BRCA2* PVs were associated with three- to fourfold increased risks of pancreatic and stomach cancers in both sexes [34], as well as with a twofold increased risk of prostate cancer, with a cumulative risk to age 80 years of 27% for male *BRCA2* PV carriers [34].

### 3.3. PALB2

*PALB2* can be considered as the third most important gene, following *BRCA1* and *BRCA2*, in terms of BC susceptibility. *PALB2* is located on the short arm of chromosome 16 and encodes for a 1186 amino acid protein. PALB2 protein has a large number of interactions with other DNA damage response proteins that function in DNA repair by HR [83].

*PALB2* PVs were initially found in 1% of families with BCs and were found to be associated with a twofold FBC risk [84,85]. Subsequent pedigree-based investigations showed that *PALB2* PVs were associated with a sevenfold increased FBC risk and a lifetime risk of about 50% (Table 1) [47]. At the population level, *PALB2* PVs showed an increased risk of about fourfold, whereas among familial FBC cases at about eightfold [23]. For male *PALB2* PV carriers, a sevenfold increased risk of BC and a cumulative BC risk of 1% to age 80 years were estimated [32,47]. In addition to BC, *PALB2* PVs were associated with two- to threefold increased risks of OC in women and pancreatic cancer in both sexes [47].

Based on the observation that *PALB2* PV carriers were fourfold more likely to have a male relative with BC than non-carriers [86], several studies have investigated the presence of *PALB2* PVs in MBC cases by candidate single-gene sequencing, and more recently by gene-panel sequencing approaches [25,27,28,29,31,32,85,87,88,89,90,91,92,93,94,95,96]. These studies showed a variable *PALB2* PV frequency, ranging from about 0.8% to 1.8%, depending on the characteristics and the size of the population analyzed [25,27,29,31,32,93].

As expected, studies enriched for high-risk MBC cases (i.e., cases with bilateral BC, and/or early onset BC, and/or a positive family history of BC) showed a higher *PALB2* PV frequency. Overall, a higher frequency of *PALB2* PVs in high-risk MBC cases than in high-risk FBC cases was observed (4% vs. 1%) [93].

Moreover, *PALB2* PVs were frequently observed in families with history of cancers other than BC/OC, including melanoma, pancreatic, prostate, and stomach cancers, suggesting that *PALB2*-related families may resemble *BRCA2*-like families, in which MBC and several other cancers may be found in addition to FBC [87,90,91,93,97,98].

There is evidence that *PALB2*-associated FBCs may display peculiar pathological features, including TNBC status [24]. However, no evidence of such association has emerged for MBC yet.

### 3.4. CHEK2

The *CHEK2* gene is located on the long arm of chromosome 22 and encodes for a 543 amino acid protein. CHEK2 protein is a tumor-suppressive serine/threonine kinase that is involved in cell cycle progression and DNA structure modification, and it is part of the DNA damage response system activated by genotoxic stress [99].

The protein truncating variant *CHEK2* c.1100delC was the first BC genetic risk factor identified after *BRCA* genes [100,101]. At population level, *CHEK2* c.1100delC showed an increased risk of about 2.5-fold, whereas among familial FBC cases was about 4.8-fold [23,24,102,103].

The *CHEK2* c.1100delC has been initially shown to confer approximately a 10-fold increase in BC risk in men, and it was estimated to account for 9% of familial high-risk MBC cases [100]. However, those results were not replicated in all populations or in unselected cases [27,32].

Notably, the contribution of the *CHEK2* c.1100delC PV to BC predisposition in both sexes varies by ethnic group and from country to country, with a founder effect in North-Eastern Europe, and a decreased frequency in North to South orientation [78,100,104,105,106,107,108].

Both single-gene direct sequencing and the introduction of NGS multigene panels have enabled the identification of additional PVs in the *CHEK2* gene, mainly missense variants. Overall, the most recent estimates confirmed that protein truncating variants in *CHEK2*, including the c.1100delC, are associated with an approximately 2.5-fold FBC risk, whereas *CHEK2* pathogenic missense variants are shown to confer a small increase in FBC risk, below twofold (Table 1) [23,24].

Data on *CHEK2* PVs besides the c.1100delC in MBC cases are still scarce. In recent studies, *CHEK2* PV prevalence ranged from 0.4% to 4.1%, and risk estimates ranged from 2.43 to 3.78, based on the population analyzed [25,26,27,29,31,32]. Our recent Italian case–control study showed no association between *CHEK2* PVs and increased MBC risk, confirming the limited role of *CHEK2* PVs in the Italian population in both sexes [32].

Female *CHEK2* PV carriers were shown to be more strongly predisposed to ER-positive BCs [23,24,109]. In other reports, *CHEK2* PVs were associated with all BC subtypes except for TNBC [24,110].

Although *CHEK2* PVs were observed in a wide range of cancer types, there are no reliable additional cancer risks associated with *CHEK2* PVs, besides BC. Evidence for association with increased risk of colorectal and prostate cancers is emerging [111,112].

### 3.5. ATM

The *ATM* gene is located on the long arm of chromosome 11 and encodes for a 3056 amino acid protein. This protein is mainly involved in cell cycle regulation and DNA damage recognition and repair [113].

*ATM* is involved in ataxia telangiectasia, a rare disease inherited in an autosomal recessive pattern. Individuals homozygous or compound heterozygous for a germline *ATM* PV develop ataxia telangiectasia. Heterozygous PVs in *ATM* are relatively common in the population, with a prevalence of about 0.35%, and they are frequently observed in cancer patients; however, the magnitude of cancer risk remains uncertain [33]. Many years ago, the seminal study of Swift and colleagues, investigating an excess risk of cancer in a series of 110 ataxia-telangiectasia families, suggested that the relative risk of cancer incidence doubled in men and tripled in women, with BC being the most associated cancer [48].

Studies investigating the role of *ATM* in FBC reported a PV prevalence ranging from 0.6% in population-based studies to 2.7% in studies enriched for familial cases. Estimates of BC risk in women heterozygous for germline *ATM* PVs ranged from a two- to fivefold increased risk, compared to women without *ATM* PVs [33,48,49,50,51,52,53,54]. Population-based risk estimates ranged from two- to threefold, with a cumulative lifetime risk of about 20–60% (Table 1) [50].

In MBC, heterozygous *ATM* PVs were found with a frequency ranging from 0.5% to 1.96% depending on the population analyzed [25,26,27,32,114]. Although larger studies are needed to better estimate the BC risk in men with *ATM* PVs, recent studies suggested that *ATM* may be considered as a possible risk gene also in MBC susceptibility with up to about fourfold increased risk [32,33].

Recently, *ATM* PVs were also associated with moderate-to-high risks (two- to fourfold) of pancreatic, prostatic, and gastric cancers, as well as with low-to-moderate risks (<2-fold) for OC, colorectal cancer, and melanoma [33].

### 3.6. Other Genes

The involvement of *BRCA1* and *BRCA*2 in the HR pathway promoted mutation screening of other DNA repair genes functionally linked to these two genes [115]. Most of these candidate BC genes have been included in multigene panels used for BC patients of both sexes. Overall, to date, *BRCA1*, *BRCA2*, *PALB2*, *CHEK2*, and *ATM* are the only well-established BC risk genes at the population level [18,116,117]. However, PVs in other genes may also increase the risk of BC, particularly for specific subtypes.

PVs in *FANCM*, a gene encoding a protein involved in the Fanconi anemia (FA) molecular pathway, have been associated with increased BC risk [118,119]. Specifically, *FANCM* PVs have been associated with a 2–4-fold increased BC risk in case–control studies conducted in different European populations [120,121]. Overall, a high frequency of protein-truncating variants in the *FANCM* gene (about 2%) was identified in *BRCA1*/2-negative BCs [122].

Notably, recent evidence has shown peculiar genotype–phenotype correlations for *FANCM*, as PVs seem to be moderate risk factors for ER-negative and TNBC. In particular, PVs within the more 5′ region of the *FANCM* gene may have a stronger effect on the risk for ER-negative BC. Conversely, the risk effects of *FANCM* PVs within the more 3′ region are probably lower [123].

*FANCM* PVs were also found in MBC at a frequency of 0.5%, which was raised up to 1% when only cases at increased genetic risk for BC were considered; however, risk estimates are not available yet [27,32,124].

PVs in OC risk genes, proposed also as candidate BC predisposing genes, such as *BARD1*, *RAD51C,* and *RAD51D*, were associated with moderate risk of ER-negative BC in women [23,125]. At present, there is no evidence that *BARD1*, *RAD51C,* and *RAD51D* PVs may contribute to BC susceptibility in men, probably due to the low frequency of the ER-negative subtype in MBC [25,27,28,32,126].

PVs in the *BRIP1* gene were originally associated with low-penetrance BC risk [127]; however, some studies indicated that *BRIP1* PV carriers have a higher risk for postmenopausal OC rather than BC [128,129,130]. Consistently, no evidence was found that germline variants in *BRIP1* might contribute to MBC predisposition [25,27,28,32,131].

Other genes initially linked to BC susceptibility, but whose association was not replicated in recent large case–control studies, include *NBN*, *BLM*, *GEN1*, FA genes other than *FANCM*, mismatch repair genes, and *RAD50*, as well as genes identified by whole exome sequencing analysis, such as *RECQL*, *RINT1*, and *XRCC2* [18,23,24,132,133].

### 3.7. Syndromic Genes

PVs in genes associated with hereditary multi-cancer syndromes have been implicated in hereditary BC, although they accounted for a very small percentage of BC cases in both sexes.

High-penetrance PVs in *TP53*, *PTEN*, and *STK11* genes, involved in Li–Fraumeni, Cowden, and Peutz–Jeghers syndromes, respectively, are known to confer a high risk of childhood and adult cancers, including BC, with lifetime risks ranging from 40 to 80% [134,135,136,137,138], although these estimates may be overestimated due to the rarity of the conditions.

PVs in *CDH1*, the hereditary diffuse gastric cancer gene, have been associated with increased BC risk, particularly of lobular histology and ER-positive status [139].

In addition, PVs in *NF1*, the gene associated with neurofibromatosis 1, may also moderately increase BC risk both in women and men [27,140].

Overall, it is unlikely that these PVs would account for a relevant proportion of BC in the absence of their respective syndromes [141,142]. Recent studies confirmed that PVs in these syndromic genes are rarely detected by multigene panel testing performed at the population level due to the rarity of the variants, early single-gene testing at younger age, and/or early death. Although these genes, particularly *TP53*, are considered bona fide BC risk genes, the debate on whether to include these genes in clinical multigene panels for use in BC patients is still open [18,143].

### 3.8. Common Low-Penetrance Risk Variants

In the last 15 years, GWAS performed by large international consortia identified more than 300 common single-nucleotide polymorphisms (SNPs) associated with BC [36,37,38,39,40,41,42,43,44,144,145,146,147,148,149,150]. These SNPs act as common low-penetrance allele variants, each generally conferring a relative BC risk < 1.40 [151,152]. Overall, these SNPs are estimated to explain about 20% of the familial risk of BC in women [152,153].

Notably, the relative risk associated with several of the loci identified shows BC subtype specificity, specifically defined by hormonal receptors status [150,153]. Associations with most of the susceptibility loci are stronger for ER-positive rather than for ER-negative BCs, probably because the majority of BC cases are ER positive [151,154]. Studies focused on ER-negative BC and TNBC identified about 20 specific loci associated with ER-negative disease, accounting for approximately 14% of familial relative risk for ER-negative BC [150,155].

A few studies addressed the role of low-penetrance variants in MBC susceptibility through GWAS or a candidate SNP genotyping approach [41,42,44,156,157,158,159,160,161]. Overall, five loci were associated with increased MBC risk. These loci were also associated with increased FBC; however, they seem to confer greater risks of BC in men than in women [41,44].

Common genetic variants could also act as modulators of the risk conferred by PVs in the high-penetrance BC susceptibility genes *BRCA1* and *BRCA2* and, more recently, also in the high-to-moderate penetrance genes *PALB2*, *CHEK2*, and *ATM* [42,144,162,163,164,165,166,167,168].

Although the relative risk associated with SNPs is low, they are likely to be responsible for a substantial percentage of hereditary and sporadic BCs due to their high frequency at the population level and to polygenic effect [35]. When summarizing all SNPs in a polygenic risk score (PRS), the cumulative risk can be substantial. Risk profiling based on a combined SNP effect can identify individuals at substantially increased or reduced BC risk, providing the basis for targeted prevention. Thus, the PRS can aid in stratifying patients into different risk categories of developing BC [169,170,171,172].

Recently, a PRS including 313 risk SNPs has been constructed and validated for the prediction of BC risk in women of European ancestry, showing that women with high PRS have the same risk as ten-year-older women with average PRS [169].

PRS models developed based on associations from FBC GWAS were also evaluated in MBC risk [44]. The recently developed 313-SNPs PRS, identified in FBC [169], was also associated with MBC risk, and its distribution in male cases was similar to that of FBC cases, suggesting a shared genetic architecture of BC in the two sexes [44].

The 313-SNP PRS was associated with both female and male BC risk in *BRCA* PV carriers, suggesting that risk profiling on the basis of PRS may provide a further individual cancer risk stratification for carriers of *BRCA1*/2 PVs, with implications for their clinical management [173,174]. Notably, distribution in male *BRCA* PV carriers was similar to female *BRCA* PV carriers but lower than in cases from the general population, possibly reflecting a general attenuation of the effect sizes of common variants on genetic risk in the presence of a PV in a high-risk gene.

It was recently demonstrated that PRS created meaningful risk gradients among female carriers of PVs in cancer-predisposing genes other than *BRCA*, including *ATM*, *CHEK2*, *PALB2*, *BARD1*, *BRIP1*, *CDH1,* and *NF1*. In particular, PRS may help differentiate BC risk among carriers of PVs in well-established moderate-penetrance genes, such as *CHEK2* and *ATM*, enabling more informed decisions about screening practices and more personalized risk management approaches [175]. Currently, no data on the possible joint effect of PVs in moderate-penetrance BC predisposition genes and PRS with MBC are available.

## 4. Implication of Gender-Oriented Genetic Testing and BC Management

### 4.1. Preventative Strategies

Individuals with suspected BC predisposition should be offered a genetic screening to identify PVs in established HBOC genes, mainly *BRCA1*/*2* [176,177].

However, currently, genetic testing remains somewhat restricted for BC patients. Based on referral criteria from genetic testing from the current available guidelines, women with TNBC, bilateral disease, or young-onset disease might be offered a genetic test at diagnosis, but most will be offered testing only if they also have a family history of the disease [177].

On the other hand, the National Comprehensive Cancer Network (NCCN) recommendations indicate that all men diagnosed with BC should be routinely screened at least for *BRCA1* and *BRCA2* PVs, regardless of age or family history, which could prove to contribute invaluable genetic information to unaffected family members [177].

Nevertheless, the assessment of the a priori probability of identifying a PV is an important component of pre-test counseling. Risk assessment models to estimate the risk of carrying a *BRCA* PV, such as BRCAPRO, have been validated for use in patients of both sexes [178,179,180].

Both men and women have the same risk of inheriting a *BRCA* PV, but men are ten times less likely to get tested [181]. However, more than half of male *BRCA* PV carriers reported that they underwent genotyping for the sake of their children or family, rather than to learn about their own cancer risk [182]. In most genotyping programs, less than 10% of the individuals tested are men [183].

It has been reported that male *BRCA1*/2 PV carriers may be under-informed about their personal cancer risk [184], thus suggesting the need of increasing awareness of possible risks of cancers, including not only BC but also common male cancers like prostate cancer, as well as rare but lethal cancers such as pancreatic and stomach cancers, for male *BRCA* PV carriers [34].

In this context, it was recently proposed to rename the HBOC syndrome with a name perceived as more gender-neutral and inclusive of the broad cancer spectrum observed in *BRCA1*/2 PV carriers [185].

Through cascade testing within high-risk families, genetic counseling and testing should be offered to healthy women and men. PVs have a large effect on lifetime risk of BC and their identification enables personalized surveillance or even risk-reducing strategies therapy for high-risk individuals (Table 2).

Women with germline PVs in *BRCA* genes may opt to an enhanced BC screening or undergo prophylactic bilateral mastectomy; primary chemoprophylaxis with tamoxifen or other selective ER modulators has also been recommended [177]. Thus far, the management guidelines for men harboring *BRCA* PVs are still often based on low-level evidence and/or expert opinion [177,186,187].

For men carrying *BRCA* PVs, the management plan involves starting breast self-exam training and education at the age of 35. As part of this approach, clinical breast exams are recommended every 12 months, starting from the age of 35. Recent studies have shown that mammography may be beneficial in selective screening of men at high risk for developing BC depicting clinically occult early stage malignancy in this population [188,189,190]. Based on this evidence, it is suggested to consider an annual mammogram for men with *BRCA2* PVs. The ideal starting age for mammograms is either 50 years or 10 years before the earliest known occurrence of MBC in the family.

Once individuals reach the age of 40, it is recommended to start prostate cancer screening for *BRCA2* carriers. For those with *BRCA1* PVs, it is advised to consider prostate cancer screening as well. Additionally, there should be contemplation of pancreatic cancer screening from the age of 50 (or 10 years younger than the earliest exocrine pancreatic cancer diagnosis in the family, whichever comes earlier). This is especially relevant for individuals with exocrine pancreatic cancer in ≥1 first- or second-degree relatives from the same side of the family (or presumed to be from the same side of the family) as the identified PVs [177]. MBC cases found to carry *BRCA1*/2 PVs are at an increased risk for developing contralateral breast and non-breast second malignancies [34,72], highlighting the need of a specific surveillance scheme for these men [191]. Notably, more than half of male *BRCA* PV carriers did not adhere to the screening guidelines recommended after disclosure of genetic test results. For example, only 25% of male *BRCA2* PV carriers reported to have annual clinical breast examination and/or perform breast self-examination [182].

In the last years, gene panels evaluating simultaneously a variable number of BC-associated genes or multiple cancer-associated genes have begun to be routinely used in clinical practice for familial BC cases, including male patients, although for most of the genes included in the multigene panels, robust evidence of association with BC risk are currently unavailable. Recommended guidelines for early detection and cancer risk reduction in women with PVs in moderate-penetrance risk genes increasing lifetime cumulative risk over 20%, including *PALB2*, *ATM,* and *CHEK2*, are starting to become available. This is not the case for male carriers of PVs in the same genes. The recent MBC risk estimates provided for *PALB2* have been instrumental in establishing guidelines for the clinical management of male carriers of *PALB2* PVs [32,34,47]. *PALB2* risk estimates for MBC are comparable, if not higher, to those recently reported for *BRCA1* [34]. In light of these results, the most recent NCCN guidelines suggested that it is reasonable to consider for male *PALB2* PV carriers a BC screening similar to that for male carriers of *BRCA1* PVs [177].

Notably, the latest NCCN Guidelines includes a new section for transgender and gender diverse people who have a hereditary predisposition to cancer, including BC [177]. Overall, given the lack of prospective data, recommendations on appropriate cancer risk reduction and/or screening options must be made on a case-by-case basis.

Regarding transgender men, gender-affirming hormone therapy with testosterone might alter the risk of BC in individuals with a hereditary susceptibility to BC, but data are still limited. Transgender men with a germline PV in a BC gene may want to consider risk-reducing mastectomy instead of gender-affirming breast surgery (top surgery), which typically retains some breast tissue and the nipple areolar complex [192]. For transgender men with a PV in a BC gene who have had top surgery, or no breast surgery, BC screening is recommended to begin at an earlier age and may include mammography and breast MRI. For individuals with a personal or family history suggestive of hereditary BC, it is recommended that genetic testing be performed prior to breast surgery to inform the type of surgery [177].

Regarding transgender women, gender-affirming hormone therapy with estrogens and anti-androgens increases breast tissue and may increase BC risk. Nonetheless, such therapy is not contraindicated, even in the presence of a PV in a BC gene. While there are limited data on BC surveillance in transgender women, NCCN guidelines suggest a BC screening like that for cisgender males at increased hereditary risk [177].

Overall, less than 10% of all BC cases are attributable to the monogenic causes sought in clinical practice. Women with familial BC for whom genetic testing did not detect a PV should be offered a personalized risk assessment based on their detailed family history, demographic risk factors, and polygenic risk, using risk assessment tools such as CanRisk [193].

Risk stratification based on PRS is about to enter clinical practice in order to further improve screening and prevention strategies for all women [194,195]. Thus, PRSs are becoming indispensable in identifying high-risk individuals who could benefit, with greater advantages, from targeted strategies according to current clinical guidelines. An integrated model, implemented in risk prediction models and including classical risk factors, family history, and PRS, would provide the highest level of risk stratification [170].

Clinical implementation of PRS may be also particularly useful to stratify risk in cohorts where there is a higher prior probability of disease, for example in carriers of PVs in BC risk genes, to personalize cancer screening. For example, the NCCN recommends magnetic resonance imaging screening for women with a lifetime BC risk > 20% [177].

Female *BRCA1* and *BRCA2* PV carriers are above the 20% threshold, and thus PRS information is unlikely to change clinical recommendations for these women. On the other hand, incorporating PRS into BC risk estimation may help identify 30% of *CHEK2* and nearly half of *ATM* carriers below the 20% lifetime risk threshold, suggesting the addition of PRS may prevent over screening and enable more personalized risk management approaches [175].

Because the age-standardized incidence of MBC is only 1/100,000 person-year with a lifetime risk of about 1/1000, there is no role for risk assessment and BC screening in the general male population. However, PRS may be applied to high-risk men, especially male *BRCA* PV carriers, which may benefit from a more refined stratification of the individual cancer risk to inform clinical management. Polygenic risk may identify male carriers of *BRCA* PVs, at both sufficiently reduced or increased risks of BC, in order to aid prevention and screening decisions [42,174].

### 4.2. Personalized Therapeutic Management

The development of NGS technologies has produced a large amount of genomic data in a wide variety of cancers, including BC. These data allowed for the identification of molecular alterations that are potential predictive biomarkers or therapeutic targets to guide personalized treatment in women with BC [196].

To date, approaches to treating men with BC have been extrapolated largely from research conducted in women with BC, although recent genomic and transcriptomic studies showed that MBCs and FBCs, including those associated with germline *BRCA1*/2 PVs, are different in the somatic landscape [71].

Overall, FBCs are characterized by a high frequency of *PIK3CA* mutations (29–45%) [197]. On the other hand, *BRCA1*-positive FBCs show the highest frequency of *TP53* mutations (up to 80%) and the lowest frequency of *PIK3CA* mutations (9%). In MBC, *PIK3CA* mutations show a high frequency (10–36%) while *TP53* mutations are at a significantly lower frequency (3–10%) compared with FBC [198,199,200]. On the other hand, *TP53* mutations were more frequently in *BRCA*-positive MBCs, and *PIK3CA* mutations were more frequently in *BRCA*-negative MBCs [69,200,201,202,203,204].

It is well established that FBC can be classified into molecular subtypes based on gene expression, with *BRCA1*-mutated tumors showing a prevalence in the basal-like subtype, while *BRCA2*-mutated FBC may show gene expression patterns that resemble those found in luminal epithelial cells [205,206,207]. Recent transcriptome data on MBC indicate that germline mutational status could impact also on MBC transcriptome profiles, defining subgroups that may be driven by different underlying molecular pathways [208]. Specifically, male breast tumors arising in patients with germline PVs in *BRCA* and *PALB2* were characterized by the activation of the cell cycle pathway, suggesting a possible use of CDK4/6 inhibitors in this setting. In addition, these tumors were characterized by a high HER2 score signaling, suggesting that they might benefit from treatment with trastuzumab [209,210].

Overall, MBC patients show a worse prognosis when compared with FBC patients [11]. Significantly reduced survival rates were also registered in the subgroup of MBC patients with *BRCA1*/2 PVs, while survival rates of FBC patients do not seem to be largely affected by *BRCA1*/2 PVs [68,211,212]. This disparity in prognosis and survival between male and female BC patients may be due to factors yet to be identified and may reflect the lack of specific management strategies in MBC.

The management of hormone-receptor-positive MBC aligns with treatment modalities utilized in female counterparts. Aromatase inhibitor (AI) combined with gonadotropin-releasing hormone agonist (GnRH-a) has become a valid treatment in FBC. However, the available data on the efficacy of this treatment in men is currently confined to case series, and there is an absence of comparative data on their efficacy [213,214].

Based on real-world data and limited studies, it was considered reasonable to extrapolate the use of additional treatment options for men with BC. These options include CDK4/6 inhibitors, mTOR inhibitors, and PI3K inhibitors, employed in conjunction with endocrine therapy. Also, in the context of metastatic BC, the use of chemotherapy, HER2-targeted therapy, and immunotherapy in men is currently guided by treatment principles analogous to those applied in women [9,215].

In the adjuvant setting with a high risk of recurrence as well as in the metastatic setting, PARPi are approved for heterozygous germline or somatic *BRCA* PV carriers with HER2-negative BC [216]. PVs in *PALB2*, *CHEK2*, and *ATM* and genomic instability scores have also been tested as additional predictive biomarkers of increased sensitivity to PARPi treatment [217]. Data suggest that BC associated with *PALB2* PVs are highly sensitive to PARPi, while no responses were observed with *CHEK2* or *ATM* PVs. The potential effectiveness of PARPi is demonstrated also in other types of *BRCA*-associated tumors including prostate cancer and OC [218,219,220,221,222]. However, little is known about the use of PARPi for the optimal management of BC in men, thus further highlighting the need to extend clinical trials to male patients, as recently suggested by the Food and Drug Administration (FDA) [9,223,224].

Immunotherapy, a promising therapeutic strategy, initially used to treat metastatic BCs and TNBCs, has a limited use in BC in general because it is considered an immunologically ‘cold’ tumor [225]. Notably, higher tumor-infiltrating lymphocyte levels have been reported in *BRCA*-associated BCs compared with sporadic BCs [226]. It was recently shown that *BRCA2* PVs may affect the tumor microenvironment differently than *BRCA1* PVs, and that *BRCA2*-associated tumors might respond better to checkpoint blockade immunotherapy [227]. Immune response recently emerged as the most relevant process able to discriminate MBC subgroups at the transcriptional level, particularly in *BRCA*-associated MBCs [208], opening for further investigation of immune-related features in a subgroup of male breast tumors.

## 5. Open Challenges and Future Directions

Current data suggest that the genetic architecture of BC predisposition, including high-, moderate-, and low-penetrance variants, is similar in both sexes; however, differences in the impact of the risk conferred by specific genetic factors, including PVs in *BRCA* genes, in *PALB2*, and in SNPs, emerged. These differences suggest that the heritable influence on BC susceptibility may be context dependent, perhaps influenced by non-genetic factors present in the breast microenvironment, including hormones, that may differently induce tumors in a mutant background [228]. However, the molecular basis of gender-specific genetic susceptibility of BC is still unknown and deserves to be investigated.

Although FBC has been the guide for MBC research to date, exploration into complex biological processes and pathogenetic mechanisms of BC might be facilitated by MBC, being rare and unencumbered by the many confounding factors that exist in FBC. These MBC characteristics offer a unique opportunity to uncover many of the interrelationships that are inherent to the disease in both genders. MBC may help uncover underlying features of BC genetics in general, as well as those shared with other *BRCA*-associated cancers, including prostate and pancreatic cancers.

In this context, the need for large-sample-size studies is quite compelling in MBC, a rare disease. To date, international studies on BC have put special efforts to include and analyze also male patients, underlining important discoveries in terms of genetic susceptibility and risk assessment, as well as making the expansion of the analysis to larger independent case series promising [174]. For this purpose, the large international Confluence project (https://confluence.cancer.gov, accessed on 20 December 2023) has been established, with the aim to study BC genetic susceptibility in women and men of multiple ancestries by integrating existing and new genome-wide genetic data across several BC consortia, including the BC Association Consortium (BCAC), the Consortium of Investigators of Modifiers of BrcA1/2 (CIMBA), and the newly established MBC genetics consortium (MERGE).

To provide a proper gender-oriented BC management, we need to address the peculiar needs of transgender and gender diverse individuals, particularly those at increased BC risk due to hereditary PVs. To date, limited scientific literature and recommendations exist on specific medical management strategies for high-risk transgender individuals [177]. The identification of a PV in a BC susceptibility gene may impact a transgender person’s decisions regarding hormonal and/or surgical transition; however, the effect of gender-affirming treatments on BC risk remains largely unexplored [229,230]. Thus, there is a need for additional research to elucidate the impact of these treatments and their interactions with BC hereditary susceptibility. Furthermore, exploring optimal preventative strategies tailored to these specific populations is essential [231].

## 6. Conclusions

Implementation in the clinical practice is the final phase of the investigation of BC susceptibility. As personalized approaches for genetic testing, risk assessment, and cancer screening are ready to enter clinical practice, current practice presents still unresolved questions and challenges, including the use of referral criteria for testing vs. population screening in women with BC, the choice of the genes to be included in gene panels, the implementation of PRS in cancer risk assessment models, the risk of tumors other than BC, germline vs. tumor testing, and the clinical management options for PV carriers [232,233]. In addition to this, evaluating the genetic component of BC from the perspective of gender medicine is mandatory to successfully implement personalized strategies. Moreover, the characterization of both germline and somatic genomic landscape may concur to establish, with greater precision, which BC patients of both sexes can benefit from targeted therapeutic strategies. Improving individualized preventative and therapeutic strategies with up-to-date genomics tools will ensure the centrality of each individual in innovative BC precision management, taking into consideration gender-specific characteristics. Overall, addressing all the challenges and the open issues regarding BC genetic predisposition will have an important clinical impact on the management of all patients towards reaching gender equality in BC care delivery.

## Figures and Tables

**Table 1:** BC risks conferred by PVs in established BC susceptibility genes in both sexes. Estimates may vary according to study population and/or study design.

	Female Breast Cancer			Male Breast Cancer		
Gene	OR	Absolute Risk	Study	OR	Absolute Risk	Study
*BRCA1*	>10	>50%	[23,24,45,46]	4	0.4%	[34]
*BRCA2*	>5	>50%	[23,24,45,46]	40	4%	[34]
*PALB2*	3.4–7.18	40–60%	[23,24,47]	7.3	0.9%	[47]
*CHEK2*	2.5	20–40%	[23,24]	2.43–3.78	na	[25,31,32]
*ATM*	2.5–5	20–30%	[33,48,49,50,51,52,53,54]	1.78–4.8	na	[31,32,33]

**Table 2:** BC management for female and male carriers of PVs in BC risk genes involved in both sexes.

	Female PV Carriers	Male PV Carriers
*BRCA1/* *BRCA2*	Education regarding signs and symptoms of cancer(s), especially those associated with *BRCA* PVs.	
	Breast awareness starting at age 18 years. Clinical breast exam, every 6–12 months, starting at age 25 years. **Screening**: Age 25–29 years, annual breast MRI screening with and without contrast (or mammogram, only if MRI is unavailable) or individualized based on family history if a BC diagnosis before age 30 is present. Age 30–75 years, annual mammogram and breast MRI screening with and without contrast. Age > 75 years, management should be considered on an individual basis. For individuals with a *BRCA* PVs who are treated for BC and have not had a bilateral mastectomy, screening with annual mammogram and breast MRI should continue as described above. **Risk reduction**: Discuss option of RRM Consider risk reduction agents as options for BC, including discussion of risks and benefits	Breast self-exam training and education starting at age 35 years. Clinical breast exam, every 12 months, starting at age 35 years. **Screening**: Consider annual mammogram, especially for those with BRCA2 PVs in whom the lifetime risk of BC is up to 7%, starting at age 50 or 10 years before the earliest known MBC in the family (whichever comes first). **Risk reduction**: not recommended.
*PALB2*	**Screening**: Annual mammogram and breast MRI with and without contrast at 30 years. **Risk reduction**: Discuss option of RRM	**Screening**: it is reasonable to consider BC screening similar to that for carriers of a *BRCA1* PVs. **Risk reduction**: not recommended.
*CHEK2*	**Screening**: Annual mammogram at age 40 years and consider breast MRI with and without contrast starting at age 30–35 years. **Risk reduction**: Evidence insufficient for RRM, manage based on family history	**Screening**: NA **Risk reduction**: NA
*ATM*	**Screening**: Annual mammogram at age 40 years and consider breast MRI with and without contrast starting at age 30–35 years. **Risk reduction**: Evidence insufficient for RRM, manage based on family history	**Screening**: NA **Risk reduction**: NA

Abbreviations: P/LP, pathogenic/likely pathogenic; MRI, magnetic resonance imaging; RRM, risk-reducing mastectomy; NA, not applicable.
